# Antimicrobial Potential of Epiphytic Bacteria Associated With Seaweeds of Little Andaman, India

**DOI:** 10.3389/fmicb.2018.00611

**Published:** 2018-04-04

**Authors:** Perumal Karthick, Raju Mohanraju

**Affiliations:** Department of Ocean Studies and Marine Biology, Pondicherry University, Port Blair, India

**Keywords:** *Alcanivorax dieselolei*, Little Andaman, Furan, *Gracilaria corticata*, seaweeds

## Abstract

Seaweeds of the intertidal regions are a rich source of surface associated bacteria and are potential source of antimicrobial molecules. In the present study, 77 epiphytic isolates from eight different algae collected from Little Andaman were enumerated. On testing for their antimicrobial activities against certain pathogens twelve isolates showed positive and six of them showed significant antimicrobial inhibition zone against *Shigella boydii* type 1, *Shigella flexneri* type 2a, *Shigella dysenteriae* type 5, Enterotoxigenic *Escherichia coli* O115, Enteropathogenic *E. coli* serotype O114, *Vibrio cholera*; O1 Ogawa*, Aeromonas hydrophila, Klebsiella pneumoniae, Staphylococcus aureus.* Based on the activity these six isolates (G1C, G2C, G3C, UK, UVAD, and Tor1) were identified by 16S rRNA gene sequence and were found to belong to the phyla Firmicutes and Proteobacteria. Purified antimicrobial compounds obtained from these isolates were identified by GC-MS. Furan derivatives were identified from G2C *Pseudomonas stutzeri* KJ849834, UVAD *Alcanivorax dieselolei* KJ849833, UK *Vibrio* sp. KJ849837, Tor1 *Exiguobacterium profundum* KJ849838. While 2-Pyrrolidinone, Phenol, 2, 4-bis (1, 1-dimethylethyl) were from G3C *Vibrio owensii* KJ849836 and (1-Allylcyclopropyl) methanol from the extracts of G1C *Bacillus* sp. KJ849835. The results of the present study shows that these six potent isolates isolated from the seaweeds are found to be a source of antimicrobial compounds.

## Introduction

Marine eukaryotes such as seaweeds are one of the primary producers which offers nutrient rich environment for microbial communities (Egan et al., 2008; Wahl, 2008). Biofilm forming bacteria isolated from the surface of seaweeds release certain compounds (Zheng et al., 2005) which serve as nutrient supplement for the algae (Croft et al., 2005), such compounds protect the host plant from the fouling communities (Rao et al., 2007). Surface associated marine organisms such as bacteria, fungi, diatoms, larval forms of marine invertebrate’s have been reported to be associated with the thallus of seaweeds (Goecke et al., 2010; Burke et al., 2011; Murthy et al., 2016; Karthick and Mohan, 2017). Such host association particularly epiphytic bacteria are sources of certain natural compounds (Singh et al., 2011; Ali et al., 2012; Martin et al., 2014). Importance of microbial diversity of seaweeds, particularly bacterial genus are highly host specific with novel species, which have emerged from these algal environment (Goecke et al., 2013).

The secondary metabolites produced by these bacteria are highly recognized for their importance in the field of biomedical applications (Armstrong et al., 2001; Kelecom, 2002; Burgess et al., 2003). Antimicrobial activity of the epiphytic bacterial communities from seaweeds have been reported (Kanagasabhapathy et al., 2006, 2008; Vijayalakshmi et al., 2008; Ravisankar et al., 2013; Horta et al., 2014; Martin et al., 2014; Karthick et al., 2015a), similarly anti-diatom activity have also been observed by Kumar et al. (2011). In the Andaman Islands, luxuriant growth of all the three groups of seaweeds are available throughout the year. Some studies on taxonomy of seaweeds have been carried out in this region but information on the epiphytic interaction and its potentiality has not been undertaken. Based on the occurrence of seaweeds in Little Andaman and their bacterial association, the present study has been undertaken to describe the isolation of epiphytic bacterial, screening, optimization, evaluation and identification of potential isolates and their antimicrobial activity against different pathogens as test organisms.

## Materials and Methods

### Isolation of Marine Bacteria

Eight different seaweeds representing all the three groups were handpicked from the intertidal region of Harminder Bay Bridge, Little Andaman, Andaman Islands, India. Among these, six species *Gracilaria corticata, Acanthophora spicifera* (red algae), *Ulva lactuca* (green algae), *Sargassum swartzii, Turbinaria ornata*, and *Padina tetrastromatica* (brown algae) are common and other two species *Mastophora rosea* (red algae) and *Caulerpa microphysa* (green algae) were found to be rare in occurrence in these islands. The collected samples were placed in sterile plastic bags and transported to the laboratory. These were washed thrice with autoclaved seawater to remove loosely bounded epiphytes, sand particles and other attached settlements on the surface of thallus. After rinsing, firmly attached epiphytic bacteria from thallus region were swabbed with sterile cotton buds and these were then swabbed on Zobell marine agar plate (Himedia). Plates were incubated for 5 days at 32°C (Lemos et al., 1985). After incubation, colonies were picked and restreaked for the isolation of individual colonies and the purity of the isolates were checked under the microscope for single morphology. These pure cultures obtained were stored at –20°C in marine broth supplemented with 20% glycerol.

### Antimicrobial Assay of Epiphytic Bacteria

The antagonistic activity of epiphytic bacteria obtained were studied on solid media by cross streaking and double-layer method described Lemos et al. (1985) and agar well diffusion method by Karthick et al. (2013b).

### Extraction of Antimicrobial Compounds

All the 77 isolates were cultured on 100ml marine broth, Luria broth and minimal media by modifying the methodology slightly by decreasing the incubation time and by increasing the temperature for obtaining better results. The culture broth was centrifuged at 10000 rpm for 30 s to remove the cells and cell free broth was extracted thrice with 100 ml of ethyl acetate. All the solvents were removed under reduced pressure at 40°C (Zheng et al., 2005). Crude extracts obtained were stored at –20°C until usage for the antimicrobial assay against targeted pathogens. Sterile media without culture being adjusted to pH 7 were used as control.

### Minimal Medium

All the potential isolates were cultured in inorganic salt medium referred to as minimal medium for the extraction of secondary metabolites (Jafarzade et al., 2013).

### Inoculum Preparation

Potential cultures were cultivated in 100 ml minimal medium supplemented with 3% NaCl, 1% glucose and 1% yeast extract as carbon and nitrogen sources in a 250 ml Erlenmeyer flask and incubated at 32°C for 24 h in an incubator shaker. Five milliliter of this culture was used as bacterial (Starter) culture (Jafarzade et al., 2013).

### Effect of pH

1 ml of starter cultures were grown with minimal media supplemented with 3% NaCl, 1% glucose, and 1% yeast extract prepared and inoculated with minimal media supplemented with 0.75% of sodium chloride, 1% of glucose and yeast extract for the production of antimicrobial compounds with various pH levels (6–8) at 32°C for 5 days. After incubation, supernatant was extracted three times with ethyl acetate (EtOAc). The sterile media without the culture adjusted to pH was used as control. The extracts were then tested for antimicrobial activity.

### Effect of Sodium Chloride Concentration

100 ml of minimal medium was dispensed into 250 ml Erlenmeyer flasks and sterilized. Yeast extract (1%) and glucose (1%) were filter sterilized and added as nitrogen and carbon sources just prior to inoculation. One milliliter of the starter culture was inoculated into the sterilized medium. Effect of salinity in the production of antimicrobial properties at various concentrations of sodium chloride ranging from 1 to 3% with constant pH of 7 at 32°C for 5 days was experimented. After incubation cell free supernatant was extracted three times with ethyl acetate (EtOAc). Sterile media without the inoculum adjusted with various concentration of sodium chloride was used as control (Jafarzade et al., 2013). The extracts were then tested for antimicrobial activity.

### Effect of Different Concentrations of Glucose and Yeast Extract

Effect of different concentration (1–3%) of glucose and yeast extract for the production of antimicrobial compound by the epiphytic bacterial isolates was studied using 1 ml of the starter culture inoculated into the minimal medium. Other parameters such as pH 7 and 0.75% sodium chloride were maintained at optimum level during the primary screening at 32°C for 5 days. After incubation supernatant was extracted three times with ethyl acetate (EtOAc). The sterile medium containing glucose, yeast extract and sodium chloride was used as control. The extracts were then tested for antimicrobial activity.

### Test Microorganisms

Eighteen bacterial pathogens namely *Escherichia coli* MTCC 443*, Klebsiella pneumoniae* MTCC 109*, Salmonella typhi* MTCC 733, *Staphylococcus aureus* MTCC 96, *Shigella flexneri* MTCC 1457, *Shigella flexneri* type2a 503004, *Shigella boydii* type 1 NK2379, *Shigella sonnei* NK4010, *Shigella dysenteriae* type 5 NK2440, Enterotoxic *E. coli* serotype 0115, Enteropathogenic *E. coli* serotype 0114, Shiga toxin producing *E. coli* serotype O157:H7 VT3, *Vibrio fluvialis* IDH 02036, *Vibrio parahaemolyticus* serovar O3: K6 K5030, *Vibrio cholera* O139, *Vibrio cholera* 01, Ogawa Eltor, *Aeromonas hydrophila* IDH1585, *Salmonella enterica* serovar typhi C6953 and three fungal strains *Aspergillus niger, Aspergillus flavus* and *Rhizopus* sp. were used for studying the antibacterial and antifungal assay.

### Determine Minimum Inhibitory Concentration of TLC Purified Metabolites

Minimum inhibitory concentration (MIC) of TLC purified metabolites of potential six isolates (G1C, G2C, G3C, UK, UVAD, and Tor1) was tested against *Klebsiella pneumoniae* and *Staphylococcus aureus* and it was determined by well diffusion assay. 50 mg of TLC purified extracts was dissolved in 1 ml DMSO. 50 and 100 μl/ml concentration of 50 mg/ml concentration of purified extracts was transferred into the well prepared (9 mm) with well cutter. Gentamicin was used as a positive control and Dimethyl sulfoxide (DMSO) was used as a negative control. Growth inhibition zone formed after the incubation was examined with measuring the diameter (mm) and results were recorded. All the assay was performed in triplicates.

### Partial Purification and GC-MS Analysis

Concentrated fractions were fractioned by Thin Layer Chromatography (TLC) using Silica gel plates with different solvents in a ratio of 2:2:1 ethyl acetate, chloroform and methanol. Bands was scraped from the plates and screened for antimicrobial assay. Active fraction was collected and analyzed by Gas Chromatography and Mass spectrometry GC-MS QP 2010 Shimadzu Corp (Japan). One μl of purified fractioned extract was loaded into the DB-5 Column with Helium as a carrier gas at a flow rate of 1 ml/min. Split Injection mode of the ratio of 1:20 was adopted. Temperature programming was from 75°C for 2 min further increased to 175°C with 15°C/min and then increased up to 280°C at the rate of 5°C/min. Sample run time was maintained upto 10 min. The peaks representing mass to charge ratio characteristic of the antimicrobial fractions were compared with those in the mass spectrum of NIST library identifying the corresponding organic antimicrobial compounds.

### Phenotypic Characterization

Phenotypic characterization of all the seventy seven bacterial isolates were identified following as described in the Bergey’s manual of systemic Bacteriology (Brenner et al., 2005).

### Molecular Identification by 16S rDNA Sequencing

Genomic DNA was prepared from the bacterial isolates by following the method of Mohandass et al. (2012). PCR amplification of 16S rRNA gene was conducted in a final volume of 25 μl with the bacterial consensus universal forward and reverse 16S rDNA primers 27F and 1492R (Lane, 1991). The reaction mixture contained 1x PCR buffer (Sigma, United States), 2.5 mM MgCl_2_, 200 μM DNTP’s, 1U of Taq DNA polymerase, 25 picomol of each forward and reverse oligonucleotide primers and approximately 20 ng of genomic DNA. The amplification profile consisted of an initial denaturation at 94°C for 3 min, followed by 35 cycles at 94°C for 1 min, 55°C for 1 min and 72°C for 1 min. This was followed by a final extension step of 72°C for 5 min. The samples were held at 4°C until further analysis. The PCR products were sequenced by an automated Sequencer (Applied Biosystems, Foster City, CA, United States) at the National Institute of Oceanography, Goa, India. The sequences were submitted to Gen Bank for which accession numbers were assigned.

### BLAST Search and Phylogenetic Analysis

The PINTAIL program (Ashelford et al., 2005) was used to check chimera formations. The partial 16S rRNA gene sequences of the potential isolates were compared with those available in the public databases. Identification upto the species level was determined by a 16S rDNA sequence similarity of more than 99% with that of the prototype sequence in GenBank. Sequence alignment and comparison were performed using the multiple sequence alignment program Clustal X 1.81 (Thompson et al., 1997). Sequences were edited manually to remove the gaps. Neighbor-joining method was employed to construct the Phylogenetic tree using MEGA4 software (Tamura et al., 2007) and the maximum likelihood method was adopted for calculating the evolutionary distance (Tamura et al., 2004).

### Bacterial Identification Based on Fatty Acid Methyl Ester (FAME)

Young pure cultures of SG107, 108, 114, 115, 120, and Tor6 were grown on Trypticase Soy Broth Agar (TSBA) for 24 or 48 h at 28°C. The Gas chromatographic analysis of whole cell fatty acid methyl ester (FAME) was performed for further identification and grouping of isolates. FAME extraction were performed using the standard procedures of extraction, purification, and methylation (Sasser, 1990). Fatty acid profiles generated were compared against an inbuilt Sherlock TSBA Library Version 6.0B [S/N 160284] (MIDI Inc., Newark, DE, United States). A similarity index of more than 0.500 was used for clustering of isolates at species level. Cellular fatty acid composition analysis was done at Regional center of Kochi, National Institute of Oceanography.

## Results

### Identification of Epiphytic Bacterial Isolates

**FIGURE 1:**
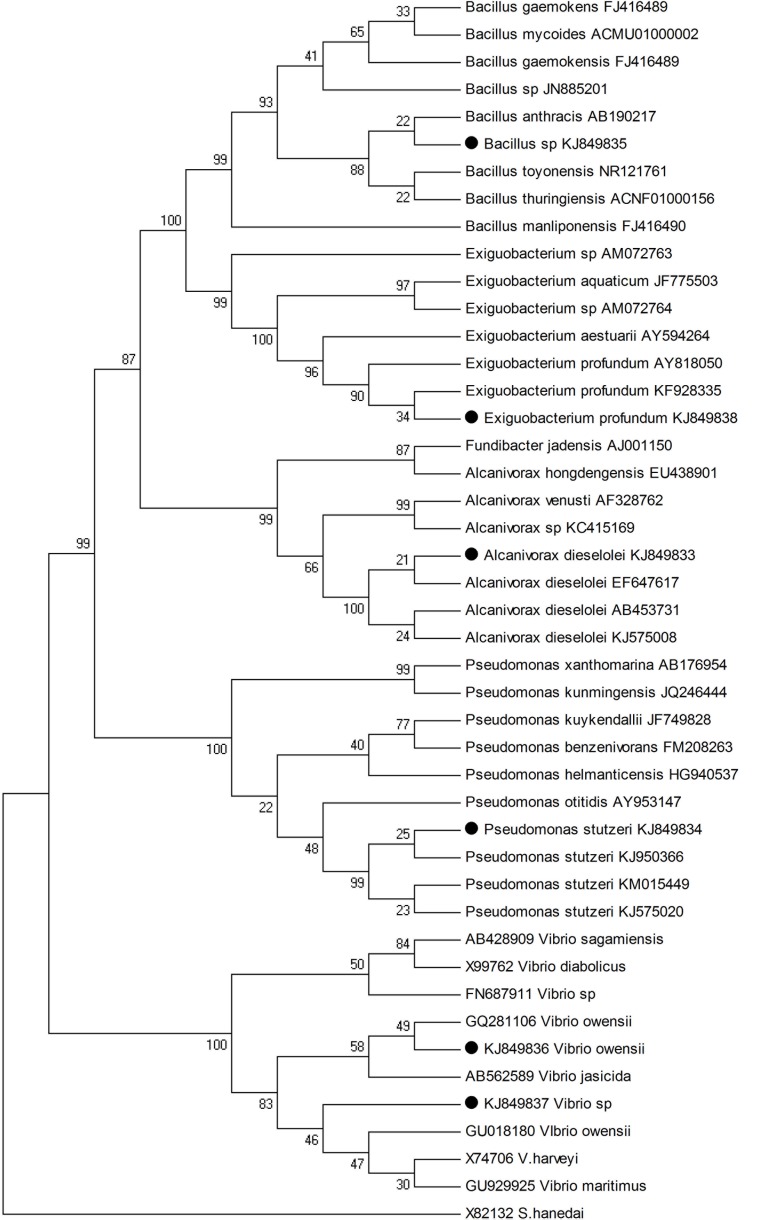
Neighbor-joining phylogenetic tree based on 16S rRNA gene sequences showing the relationship of six potential strains with its closest neighbors. Bootstrap values (50%) are shown at branch points in value and *S. hanedai* X82132 were used as outgroup.

All the seventy seven cultivable epiphytic bacterial isolates obtained from the thallus of eight different seaweeds were plated on Marine agar. These isolates were purified and based on by phenotypic characterization were assigned to belong to the phylum Firmicutes and Proteobacteria (**Table 1**). Among these isolates, six of them (G1C, G2C, G3C, UK, UVAD, and Tor1) showed wide range of activities against pathogens with an range of 10–30 mm zone of inhibition and were identified by partial 16S rRNA gene sequences (**Table 2**). The isolate G1C showed 99% similarity as *Bacillus* sp., G2C as *Pseudomonas stutzeri*, G3C and UK were identified as *Vibrio owensii* and *Vibrio* sp., respectively. The Isolate UVAD was identified as *Alcanivorax dieselolei* with 99% similarity and Torl strain was identified with 99.7% similarity as *Exiguobacterium profundum* (**Figure 1**).

**Table 1:** List of bacterial isolates obtained from different seaweeds.

S. no	Name of seaweeds	Number of isolates obtained
	**Red algae**	
1	*Gracilaria corticata* (J. Agardh) Agardh 1852	9
2	*Acanthophora spicifera* (M.Vahl) Borgesen 1910	11
3	*Mastophora rosea* (C.Agardh) Setchell 1943	6
	**Green algae**	
4	*Ulva lactuca* Linn 1753	14
5	*Caulerpa microphysa* (Weber van Bosse) J. Feldman	6
	**Brown algae**	
6	*Sargassum swartzii* (Turner) C. Agardh 1820	14
7	*Turbinaria ornata* (Turner) J. Agardh 1848	7
8	*Padina tetrastromatica* Hauck 1887	10

**Table 2:** 16S rRNA gene sequence identity of six potential bacterial isolates obtained from different seaweeds.

Isolate	Seaweed	Accession number	Identified bacteria	% identity	Phylum
G1C	*Gracilaria corticata*	KJ849835	*Bacillus* sp.	99	Firmicutes
G2C	*Gracilaria corticata*	KJ849834	*Pseudomonas stutzeri*	99	Proteobacteria
G3C	*Gracilaria corticata*	KJ849836	*Vibrio owensii*	100	Proteobacteria
UK	*Mastophora rosea*	KJ849837	*Vibrio* sp.	99	Proteobacteria
UVAd	*Ulva lactuca*	KJ849833	*Alcanivorax dieselolei*	100	Proteobacteria
Tor1	*Turbinaria ornata*	KJ849838	*Exiguobacterium profundum*	100	Firmicutes

Six other isolates showed moderate to less activity activities against the pathogens with a range of 5–10 mm zone of inhibition and were identified by FAME analysis. Among them three isolates SG107 as *Bacillus* sp., SG108 *Paenibacillus lentimorbus* and SG115 as *Bacillus sphaericus* belonged to phylum Firmicutes. The other three isolates *Pantoea agglomerans*, SG 120 was *Vibrio aestuarianus* and TR was identified as *Klebsiella pneumoniae ozaena* and were assigned to phylum Proteobacteria, SG114 (**Table 3**).

**Table 3:** Comparison of cellular fatty acid composition of 6 moderate activity identified from brown algae (1) SG107 *Bacillus* sp, (2) SG108 *Paenibacillus lentimorbus*, (3) SG114 *Pantoea agglomerans*, (4) SG115 *Bacillus sphaericus*, (5) SG120 *Vibrio aestuarianus*, (6) Tor6 *Klebsiella pneumoniae ozaenae*.

Fatty acid	SG107	SG108	SG114	SG115	SG120	Tor6
**Straight-chain saturated**						
14:00	1.99	3.03	0.81	0.75	5.10	9.31
15:00	-	-	-	-	-	-
16:00	8.01	16.57	35.53	1.62	18.34	31.44
17.00	-	-	0.27	-	0.44	-
18:00	1.08	2.48	1.40	0.29	1.33	0.78
**Terminally branched saturated**						
13:0 iso	0.28	0.43	-	0.11	0.07	-
14:0 iso	8.56	4.58	-	5.67	-	-
15:0 iso	27.43	22.96	-	52.57	0.08	-
16:0 iso	6.11	2.93	-	10.20	0.19	-
17:0 iso	4.50	5.57	0.13	3.75	-	-
18:0 iso	0.28	0.28	-	-	-	-
19:0 iso	-	-	0.51	-	-	0.50
15:0 anteiso	26.59	24.25	-	9.39	-	-
17:0 anteiso	5.57	4.65	-	2.98	-	-
**Mono-unsaturated**						
16:1w7c alcohol	2.11	0.69	-	6.43	-	-
16:1w11c	4.55	6.41	-	1.71	-	-
Sum of 18:2 w6,9c/18:0 ante	-	0.3	-	-	-	-
Sum of 18:1 w7c	-	0.1	-	-	0.08	-
Sum of 18:1w9c	-	0.20	-	0.16	0.31	-

### Antimicrobial Activity of Epiphytic Bacteria

Antimicrobial activity for all the 77 bacterial isolates were tested by adopting three different methods (Agar overlay, cross streaking and agar well diffusion technique) against 21 pathogens. Among these larger zones of inhibition were observed in agar well diffusion assay and this assay was chosen for further antimicrobial activity test. All the isolates were cultured in three different media (marine broth, luria broth and minimal medium) among the medium used minimal media showed broad range of antimicrobial activity (**Table 4**). Based on the preliminary activity only six potential isolates (G1C, G2C, G3C, UK, UVAD, and Tor1) were optimized in minimal medium for the production of antimicrobial compounds.

**Table 4:** Antimicrobial activity of six potential epiphytic bacterial isolates.

Clinical pathogens	G1C	G2C	G3C	UK	UVAD	Tor1
*Shigella boydii* type 1	+	++	++	+++	+++	++
*Shigella sonnei*	+	++	++	-	+++	++
*Shigella flexneri*	++	-	-	+++	++	++
*Shigella flexneri* type 2a	++	++	++	+++	++	++
*Shigella dysenteriae* type 5	-	++	++	+++	+++	+++
*Salmonella typhi*	+	+	-	+	++	++
*Salmonella enterica* serovar typhi	+	++	+++	+	++	+
*Escherichia coli*	++	+	+	++	-	-
Enterotoxigenic *Escherichia coli* O115	++	++	++	+++	++	+
Enteropathogenic *Escherichia coli* serotype O114	++	++	++	+++	++	+
Shiga toxin *Escherichia coli*O157:H7	+	++	++	-	++	+
*Aeromonas hydrophila*	+	++	++	+++	++	+
*Vibrio cholera*; O1 Ogawa	++	++	++	-	++	++
*Vibrio cholera* O139	-	-	-	-	-	-
*Vibrio fluvialis*	-	-	-	++	+	-
*Vibrio parahaemolyticus* serovar O3: K6	-	-	-	++	-	-
*Klebsiella pneumoniae*	+	+	++		+++	+++
*Staphylococcus aureus*	+	+	++	+++	+++	++

*(+) >10 mm, (++) 10–20 mm, (+++), 20–30 mm, (-) No inhibition zone observed.*

### Zone of Inhibitory Activity With Optimized Bacterial Isolates

Isolate G1C showed strong inhibitory activity against *Shigella boydii* (31 mm), Enterotoxigenic *E. coli* (28 mm), Enteropathogenic *E. coli* and *Aeromonas hydrophila* (23 mm) and this higher inhibition zones were obtained with minimal media supplemented with 1 and 2% Sodium chloride, 1% of glucose and yeast extract with pH in the range of 7–8. Isolate G2C was more effective against Shigatoxin *E. coli* (26 mm), *Aeromonas hydrophila* and *Salmonella typhi* (24 mm) observed from minimal medium containing only 1% of sodium chloride, glucose and yeast extract with pH in the range of 6–7. Isolate G3C was effective against *Salmonella enterica* serovar *typhimurium* (26 mm), *Vibrio cholerae* Eltor and *Shigella dysenteriae* (24 mm) and antimicrobial activity was observed with minimal media with 1 and 2% of sodium chloride, glucose and 1% of yeast extract with pH in the range of 6–7. Isolate TOR1 exhibited broad range of antibacterial activity against *Klebsiella pneumoniae*, *Shigella dysenteriae* (31 mm), *Shigella sonnei* (25 mm). UVAD isolate showed maximum zone of inhibitory activity against *Staphylococcus aureus* (30 mm), *Salmonella enterica* serovar *typhimurium* (29 mm) and *Shigella dysenteriae* (27 mm) and isolate UK displayed maximum zone of inhibition against *Aeromonas hydrophila* (29 mm), *Shigella flexneri* 2A and *Shigella flexneri* (24 mm), respectively. All these six bacterial isolates showed maximum inhibitory activity against most of the tested pathogens (**Table 4**).

### Identification of Compounds From Purified Extract

Cell free supernatant of the six potential isolates (G1C, G2C, G3C, UK, UVAD, and Tor1) that showed antimicrobial activity were purified by TLC. Results of minimum inhibitory concentration of TLC purified metabolites of all the six potential isolates at 50 mg/mL concentration diluted in 50 and 100 μl/ml concentration showed the positive results against *K. pneumoniae* and *S. aureus*. 50 μl/ml concentration of potential extracts exhibited inhibitory activity against the tested pathogens. Maximum zone of inhibition 10 mm was measured against *K. pneumoniae* by UK, UVAD, Tor1, G1C, and G3C extract at 100 μl concentration. 9 mm zone of inhibition was measured against *S. aureus* by 100 μl concentration of G2C, G3C, UK, and Tor1. Based on the inhibitory growth of bacteria at 50 μl concentration of purified metabolites, it can be concluded that minimum inhibitory concentration was observed at 2.5 mg/mL. Based on the activity purified compounds obtained from these six isolates were characterized and identified. Furan derivatives were found to be present in four of the isolates namely G2C *Pseudomonas stutzeri*, Tor1 *Exiguobacterium profundum*. UVAD *Alcanivorax dieselolei* and UK *Vibrio* sp. While 2-Pyrrolidinone, Phenol, 2, 4-bis (1, 1-dimethylethyl) were identified from the isolate G3C *Vibrio owensii* and (1-Allylcyclopropyl) methanol from G1C *Bacillus* sp.

## Discussion

Seaweed biomass were found in large quantities in both intertidal and subtidal regions of all the regions of Andaman Island and in Little Andaman’s (Karthick et al., 2013a,b). Good hemolytic activity in certain seaweeds of these Island has been reported recently (Punnam Chander et al., 2014). Besides these studies Karthick et al. (2015b) had reported on antimicrobial activity of certain seaweeds against pathogenic bacterial and fungal stains. Several authors suggested that macro algal associated bacteria were found to be an efficient producer of antimicrobial compounds (Burgess et al., 1999; Lee et al., 2006; Kanagasabhapathy et al., 2008; Karthick et al., 2015a; Ismail et al., 2016). On the other hand, certain brown algae also produced biologically active compounds which inhibited the settlement of bacterial colonies on the thallus (Nagayama et al., 2002).

In the present study it was observed that higher number of epiphytic bacteria were isolated from brown and red algae, certainly the proportion of higher isolates were from brown rather than green and red algae. On surface colonization non-pigmented bacterial isolates were found dominant in most of the seaweeds used in this study. Epiphytic bacteria from marine macro algae have been well studied in reference to their ecological importance with host organisms (Croft et al., 2005) with a dominance of Gram-negative bacteria. Similarly in the present study 46 Gram-negative bacterial isolates were isolated in comparison to 31 being Gram-positive. Bacteria belonging to genus the *Bacillus* were dominant with 20 isolates followed by other genus such as *Vibrio*, *Aeromonas*, and *Pseudomonas*.

Ravisankar et al. (2013) observed that the surface of the brown algae *Hypnea valentiae* and *Padina* *tetrastromatica* contained more number of non-pigmented bacterial colonies which are similar to our studies wherein 10 isolates were obtained from Padina *tetrastromatica*. Similar observations were observed in Tunisian waters, where 17 isolates were obtained from the thallus of *Ulva intestinalis* (Ali et al., 2010) and 10 isolates were reported from *Ulva lactuca* in Fiji waters, of which majority of the isolates were efficient antimicrobial producers (Kumar et al., 2011).

In the present study twelve isolates (15.7%) of the total 77 isolated exhibited antimicrobial activity and six isolates showed broad spectrum of activity against both bacterial and fungal pathogens. Similarly (Jayanth et al., 2002) isolated 14.52% of associated bacteria from the red algae *Gracilaria* with antagonistic properties against certain human pathogens. On the other hand 11% of associated bacteria isolated from seaweeds were reported to have antagonistic nature against *Bacillus subtilis, E. coli, S. aureus, Agrobacterium tumefaciens*, and *Saccharomyces cerevisiae* (Zheng et al., 2005).

The 16S rRNA sequences of bacterial isolates obtained from the surface of green algae *Ulva australis* and *Delisea pulchra*, belonged to the representative’s classes of *Alpha* and *Gammaproteobacteria* and interestingly *Actinobacteria, Firmicutes*, and *Bacteroidetes* were observed as antimicrobial producers (Penesyan et al., 2009). Ali et al. (2012) on 16S rRNA sequence of the isolates obtained from the surface of coralline red algae *Jania rubens* found them belong to the group Proteobacteria. Similar observation made by Singh et al. (2015) also highlighted that bacterial isolates belonged to the order *Bacillales, Pseudomonadales, Alteromonadales*, and *Vibrionales* were dominant in green algae *Ulva lactuca, U. fasciata*, and red algae *Gracilaria corticata* and *G. dura*. In this study also it’s evident that all the 77 bacterial isolates were closely related to the phylum Proteobacteria and Firmicutes. These findings substantiate that these groups are more specific to the macro algal surface. Similarly species belonging to the genera *Bacillus* and *Vibrio* were found to be strong antimicrobial producers colonizing more on the surface of seaweeds.

Genus *Bacillus* predominantly colonizes on the surface of marine niche and several studies have been reported *Bacillus* from different marine sources particularly associated with brown algae (Thakur and Anil, 2000) and from the thallus surface of different red algae (Kanagasabhapathy et al., 2008). Apart from their association with seaweeds *Bacillus* were previously isolated from sediments and seaweeds with antimicrobial properties (Prieto et al., 2012). So far, more than 800 metabolites have been reported with various biological activities from the *Bacillus* genera. Recently, cell free supernatant extracted from *Bacillus* associated with a nematode were found to be very effective against multidrug resistant *Staphylococcus aureus* (Susilowati et al., 2015). As observed in the present study one potential isolate G1C identified as *Bacillus* sp. showed remarkable activity against most of the tested pathogens, in particular against toxin producing pathogens like *Shigella boydii*, Enterotoxigenic *E. coli*, Shigatoxin *E. coli* Enteropathogenic *E. coli*, and *Aeromonas hydrophila* etc. Similarly SG107 *Bacillus* sp. and SG115 *Bacillus sphaericus* obtained from the brown algae *Sargassum swartzii* also showed moderate to less activity against few pathogens.

*Vibrios* being truly marine and they are widespread in various marine niches and are known to produce secondary metabolites for their survival. Earlier genus *Vibrio* sp., *Pseudomonas* sp., and *Bacillus pumilus* were reported to be a probiotic bacteria used in aquaculture (Hill et al., 2009). Kanagasabhapathy et al. (2008) reported that Vibrio strain isolated from red algae showed certain biological activities. In the present study, two potential *Vibrio* isolates GC3 *Vibrio owensii* and UK *Vibrio* sp. were obtained from the surface of red algae *Gracilaria corticata* and *Mastophora rosea*, respectively, and these isolates exhibited wider range of antimicrobial activity against most of the tested pathogens like *Salmonella typhi, Shigella dysenteriae, Vibrio cholerae*, and *Staphylococcus aureus*. Similarly, Pawar et al. (2015) extracted antibacterial compounds from marine *Vibrio* sp. which were found to be active against numerous pathogens.

In our study of 14 bacterial isolates were obtained green algae *Ulva lactuca* among these one isolate UVAD *Alcanivorax dieselolei* was found to possess higher range of antimicrobial activity. Previously this species *Alcanivorax dieselolei* has been reported to be isolated from the deep sea sediment involved in degrading alcanes (Liu and Shao, 2005), and petroleum products (Brito et al., 2006). Ali et al. (2010) reported that two epiphytic bacteria obtained from green alga *U. intestinalis* showed potent antimicrobial activity. These studies suggest that green algae *Ulva* species attracts novel bacterial colonization on their surface with potential microbial communities and these isolates produce various compounds to protect the host from the predators and other micro and macro fouling colonization.

*Pseudomonas stutzeri* has been reported to have wide range of biological activity by the production of secondary metabolites. Previously *Pseudomonas stutzeri* isolated from fish gut exhibited antimicrobial activity (Uzair et al., 2008), hydrocarbon degradation (Vazquez et al., 2009), and reported as uncommon opportunistic pathogen (Park et al., 2013), controlling biofilm formation (Wu et al., 2016). In this study *Pseudomonas stutzeri* isolated from the red algae *Gracilaria corticata* produced antimicrobial compounds which showing potent activity against numerous toxin producing pathogens *S. aureus, Shigella boydii, S. flexneri 2A, S. dysenteriae, K. pneumoniae, Et. E. coli, St. E. coli, V. cholerae* Eltor, *A. hydrophila.*

Earlier *Exiguobacterium* sp. showed antimicrobial properties (Shatila et al., 2013) and this bacterium also known to produce antifouling compound and thus protected the host organisms from fouling communities (Jain et al., 2013). In the present study Tor1 *Exiguobacterium profundum* isolate obtained from *Turbinaria ornata*, showed antibacterial activity against clinical pathogens *S. aureus*, *Shigella boydii, S. flexneri, S. flexneri 2A, S. dysenteriae, K. pneumoniae, Et. E. coli*, and *St. E. coli.* The same genus was identified in earlier studies from different seaweeds occurring in different geographical locations showing various biological activities. In the present study 6 potential isolates obtained from seaweeds were found to be good antimicrobial producers. The same genus was identified in earlier studies from different seaweeds occurring in different geographical locations showing various biological activities (**Table 5**).

**Table 5:** Biological activities of associated bacteria isolated from seaweeds.

Seaweeds	Associated bacteria	Biological activity	Reference
*Jania rubens*	*Bacillus firmicutes*	Antimicrobial	Ali et al., 2012
*Plocamium telfairiae*	*Bacillus anthracis*	Antimicrobial	Kanagasabhapathy et al., 2008
*Gelidium amansii*	*Bacillus cereus*	Antimicrobial	Kanagasabhapathy et al., 2008
*Grateloupia filicina*	*Bacillus cereus*	Antimicrobial	Kanagasabhapathy et al., 2008
*Porphyra yezoensis*	*Bacillus pumilus*	Antimicrobial	Kanagasabhapathy et al., 2008
*Lomentaria catenata*	*Bacillus clausii*	Antimicrobial	Kanagasabhapathy et al., 2008
*Chondrus ocellatus*	*Bacillus pumilus*	Antimicrobial	Kanagasabhapathy et al., 2008
*Laminaria saccharina*	*Bacillus* sp.	Antibacterial	Wiese et al., 2009
*Sargassum thunbergii*	*Bacillus* sp.	Antimicrobial	Zheng et al., 2005
*Ulva lactuca*	*Bacillus* sp.	Antidiatom	Kumar et al., 2011
*Padina pavonica*	*Bacillus pumilus*	Antimicrobial	Ismail et al., 2016
*Sargassum* sp.	*Bacillus pumilus*	Antibacterial	Susilowati et al., 2015
*Gracilaria corticata*	*Bacillus* sp.	Antimicrobial	In this study^∗^
*Plocamium telfairiae*	*Vibrio* sp.	Antimicrobial	Kanagasabhapathy et al., 2008
*Ulva lactuca*	*Vibrio*	Antidiatom	Kumar et al., 2011
*Stoechospermum polypodioides*	*Vibrio* sp.	Antifouling	Jain et al., 2013
*Laminaria saccharina*	*Vibrio* sp.	Antibacterial	Wiese et al., 2009
*Stoechospermum polypodioides*	*Vibrio* sp.	Antifouling	Jain et al., 2013
*Ulva reticulata* sp.	*Vibrio* sp.	Antifouling	Harder et al., 2004
*Gracilaria corticata*	*Vibrio owensii*	Antimicrobial	In this study^∗^
*Mastophora rosea*	*Vibrio* sp.	Antimicrobial	In this study^∗^
*Laminaria saccharina*	*Pseudomonas* sp.	Antibacterial	Wiese et al., 2009
*Padina pavonica*	*Pseudomonas* sp.	Antimicrobial	Ismail et al., 2016
*Sargassum sp*	*Pseudomonas koreensis*	Antioxidant	Pawar et al., 2015
*Chaetomorpha media*	*Pseudomonas argentinensis*	Antioxidant	Pawar et al., 2015
*Ulva reticulata*	*Pseudomonas stutzeri*	Antimicrobial	Dhanya et al., 2016
*Gracilaria corticata*	*Pseudomonas stutzeri*	Antimicrobial	In this study^∗^
*Stoechospermum polypodioides*	*Exiguobacterium* sp.	Antifouling	Jain et al., 2013
*Turbinaria ornata*	*E. profundum*	Antimicrobial	In this study^∗^
*Ulva lactuca*	*Alcanivorax dieselolei*	Antimicrobial	In this study^∗^

*^∗^Indicates the species identified in this study.*

In earlier studies Furan derivatives were reported to have antimicrobial properties (Kirilmis et al., 2009; Joshi et al., 2010; Ramasamy and Balasubramanian, 2012), cytotoxic agent (Wang et al., 2008) and were observed to have a wide range of biological activities like antiproliferative, antiviral, antifungal, immunosuppressive, anti-platelet, anti-oxidative, insecticidal, anti-inflammatory, anti-feedant, and cancer preventative activity (Venkateshwarlu et al., 2013). In our present study we have identified Furan compounds from four potential isolates (G2C, UVAD, Tor1, and UK). Apart from antimicrobial properties, these compounds are being used for other pharmacological properties (Bober et al., 2012). In this study G3C *Vibrio owensii* produced antimicrobial compounds such as 2-Pyrrolidinone, Phenol, 2, 4-bisdimetyl ethyl)-ester and Pyrrolo [1,2-a] pyrazine-1,4-dione. Earlier, these compounds were reported to have antimicrobial properties (Sutariya et al., 2012; Khatiwora et al., 2013; Padmavati et al., 2014; Dhanya et al., 2016). Marine *Vibrio* sp. is highly capable of producing Phenol, pyrrolo [1,2-a]pyrazine-1,4-dione, Pyrrolidinone derivative compounds containing pharmacological properties (Pawar et al., 2015). Based on earlier findings and as observed in the present study Vibrios are efficient producer of Phenol and Pyrrolidinone derivatives. In conclusion based on the findings of the present study, the compounds produced from six potential isolates (G1C, G2C, G3C, UK, UVAD, and Tor1) having effective antimicrobial properties, could be further studied for other activities. These isolates could prove to be potential candidates for the production of novel antimicrobial compounds in order to control the pathogens.

## Author Contributions

PK designed the work, performed all the experiments, analyzed and wrote the manuscript. RM contributed the designation of research work and evaluated the manuscript.

## Conflict of Interest Statement

The authors declare that the research was conducted in the absence of any commercial or financial relationships that could be construed as a potential conflict of interest.
